# The R21C Mutation in Cardiac Troponin I Imposes Differences in Contractile Force Generation between the Left and Right Ventricles of Knock-In Mice

**DOI:** 10.1155/2015/742536

**Published:** 2015-04-16

**Authors:** Jingsheng Liang, Katarzyna Kazmierczak, Ana I. Rojas, Yingcai Wang, Danuta Szczesna-Cordary

**Affiliations:** Department of Molecular & Cellular Pharmacology, University of Miami Miller School of Medicine, Miami, FL 33136, USA

## Abstract

We investigated the effect of the hypertrophic cardiomyopathy-linked R21C (arginine to cysteine) mutation in human cardiac troponin I (cTnI) on the contractile properties and myofilament protein phosphorylation in papillary muscle preparations from left (LV) and right (RV) ventricles of homozygous R21C^+/+^ knock-in mice. The maximal steady-state force was significantly reduced in skinned papillary muscle strips from the LV compared to RV, with the latter displaying the level of force observed in LV or RV from wild-type (WT) mice. There were no differences in the Ca^2+^ sensitivity between the RV and LV of R21C^+/+^ mice; however, the Ca^2+^ sensitivity of force was higher in RV-R21C^+/+^ compared with RV-WT and lower in LV- R21C^+/+^ compared with LV-WT. We also observed partial loss of Ca^2+^ regulation at low [Ca^2+^]. In addition, R21C^+/+^-KI hearts showed no Ser23/24-cTnI phosphorylation compared to LV or RV of WT mice. However, phosphorylation of the myosin regulatory light chain (RLC) was significantly higher in the RV versus LV of R21C^+/+^ mice and versus LV and RV of WT mice. The difference in RLC phosphorylation between the ventricles of R21C^+/+^ mice likely contributes to observed differences in contractile force and the lower tension monitored in the LV of HCM mice.

## 1. Introduction

The pump function of the heart is achieved by a sequence of alternating contraction and relaxation of the heart muscle. Although both the left (LV) and right (RV) ventricles contract simultaneously, there are functional and structural differences between them. Since the LV is responsible for pumping blood throughout most vessels in the body, it generates more pressure and therefore it is thicker than the RV. A normal LV has a near conical geometry with its long-axis directed from apex to base and irregular endocardial surface due to the presence of papillary muscles and trabeculae [1]. Several studies using experimental mechanics and computational modeling have proved that the helical geometry of the muscle fibers of the LV changes gradually from right-handed in the subendocardium to left-handed in the subepicardium, which produces a distinctive counter directional movement of the fiber layers in a beating heart [2–5]. The RV is less muscular because it operates at a lower pressure compared to the LV and pumps blood through the shorter distance to the lungs. In addition, the RV does very little work against gravity. The stroke work for RV is approximately 25% of that for LV because of low resistance of the pulmonary vasculature. Morphologically, the RV is distinguished from the LV by having coarser trabeculae and a lack of fibrous continuity between its inflow and outflow valves [6, 7].

Despite efforts by many, the contractile differences between the RV and LV in the healthy and/or diseased heart are poorly understood. For example, no differences were observed in the stress development, twitch duration, work performance, or shortening power between the RV and LV in dogs [8, 9]. On the other hand, small differences were observed in the contractile performance and growth of the LV and RV myocytes in dilated cardiomyopathy [10] and in myofilament function in congestive heart failure [11]. Likewise, molecular analysis of the myocardial tissue of the explanted heart of familial hypertrophic cardiomyopathy (HCM) patients showed similar mRNA and *β*-MHC protein expression levels in both ventricles but the hypertrophic phenotype was only observed in the LV [12].

This report aimed to examine the functional differences between the LV and RV at the level of papillary muscle fibers from the knock-in (KI) mice expressing the HCM-linked R21C (arginine to cysteine) mutation in cardiac troponin I (cTnI) shown to cause a malignant HCM phenotype [13]. The animal model of R21C-HCM was produced and characterized previously [14, 15]. Here we show a significant functional difference in contractile force generation between the LV and RV in the disease model expressing the R21C cTnI mutation which is not present in wild-type (WT) control mice. The presence of the effect of the mutation only in the LV is the testimony to the power of the mutation exerting detrimental effects on the function of the LV.

## 2. Materials and Methods

All animal studies were conducted in accordance with institutional guidelines. The University of Miami has an Animal Welfare Assurance (A-3224-01, effective November 23, 2011) on file with the Office of Laboratory Animal Welfare (OLAW), National Institutes of Health. The mouse model of R21C-HCM was generated and characterized earlier [14]. Seven nine-month-old homozygote R21C^+/+^ KI mice were used in the experiments and the results were compared to those of age and gender matched WT controls.

### 2.1. Histopathological Characterization

After euthanasia, the hearts from ~7-month-old WT and R21C^+/+^ KI mice were excised and immersed in 10% buffered formalin. Slides of whole mouse hearts were prepared at the Histology Laboratory (University of Miami Miller School of Medicine, Miami, FL). Paraffin-embedded longitudinal sections of whole mouse hearts stained with hematoxylin and eosin (H&E) and Masson's trichrome were examined for overall morphology and fibrosis using a Dialux 20 microscope, 40/0.65 NA (numerical aperture) Leitz Wetzlar objective, and an AxioCam HRc (Zeiss) as described previously [16, 17].

### 2.2. Preparation of Glycerinated Left and Right Ventricular Muscle Strips

The papillary muscles of the left and right ventricles from flash frozen hearts of R21C^+/+^ KI mice were isolated, dissected into muscle bundles in the buffer containing pCa 8 solution (10^−8^ M [Ca^2+^], 1 mM free [Mg^2+^] (total MgPr (propionate) = 3.88 mM), 7 mM EGTA, 2.5 mM [Mg-ATP^2−^], 20 mM MOPS, pH 7.0, 15 mM creatine phosphate, and 15 units/mL of phosphocreatine kinase, ionic strength = 150 mM adjusted with KPr), 15% glycerol, and 30 mM BDM. Solutions of increasing Ca^2+^ concentrations from pCa 8 ([Ca^2+^] = 10^−8^ M) to pCa 4 ([Ca^2+^] = 10^−4^ M) were prepared based on the “pCa-Calculator” program developed by Dweck et al. [18]. Muscle bundles were skinned in 50% pCa 8 solution and 50% glycerol containing 1% Triton X-100 for 24 hr at 4°C. Muscle bundles were then transferred to the same solution without Triton X-100 and stored at −20°C for experiments within ~5 days [19].

### 2.3. Steady-State Force Development

Small muscle strips of approximately 1.4 mm in length and 100 *μ*m in diameter were isolated from a batch of glycerinated skinned mouse papillary muscle bundles and attached by tweezer clips to a force transducer. The strips were placed in a 1 mL cuvette and freshly skinned in 1% Triton X-100 dissolved in pCa 8 buffer for 30 min. The sarcomere length was adjusted to ~2.1 *μ*m and the maximal steady-state force was measured in pCa 4 solution (composition is the same as pCa 8 buffer except the [Ca^2+^] = 10^−4^ M). Maximal tension readings (in pCa 4) were taken before and after the force-pCa curve, averaged, and expressed in kN/m^2^. The cross-sectional area of the muscle strip was assumed to be circular [16].

### 2.4. The Ca^2+^ Dependence of Force Development

After determination of initial steady-state force, muscle strips were relaxed in pCa 8 buffer and exposed to solutions of increasing Ca^2+^ concentrations from pCa 8 to pCa 4 [18]. The level of force was measured in each “pCa” solution. Data were analyzed using the Hill equation [20], where “[Ca^2+^]_50_ or pCa_50_” is the free Ca^2+^ concentration which produces 50% of the maximal force and *n*_H_ is the Hill coefficient. The pCa_50_ represents the measure of Ca^2+^ sensitivity of force and the *n*_H_ is the measure of myofilament cooperativity.

### 2.5. Passive Force Measurements

The measurement of passive force (in pCa 8 solution) in response to muscle stretch was performed as described in [16]. Briefly, after skinning the strips were washed in the relaxing solution and their length was adjusted to remove the slack. This procedure resulted in sarcomere length (SL) of ~2.1 *µ*m as judged by the first order optical diffraction using a He–Ne laser [21, 22]. This point was set as zero for both the passive force and starting length of the muscle strip. Then, the strips were stretched by 10% of its length ×4 consecutive times, and the passive force per cross-section of muscle (in kN/m^2^) was determined.

### 2.6. Analysis of Protein Phosphorylation

Flash frozen LV and RV from all groups were homogenized in CMF buffer consisting of 5 mM NaH_2_PO_4_, 5 mM Na_2_HPO_4_ (pH 7.0), 0.1 mM NaCl, 5 mM MgCl_2_, 0.5 mM EGTA, 5 mM ATP, 5 nM microcystin, 0.1% Triton X-100, 20 mM NaF (phosphatase inhibitor), 5 mM DTT, and 1 *μ*L/mL protease inhibitor cocktail. The samples were homogenized for 2 min in a Mixer-Mill MM301 at 30 Hz, chilled on ice, and homogenized again for 2 min. Homogenates were then centrifuged for 4 min at 1800 g and the supernatants were discarded. The pellets were resuspended in the CMF buffer and the myofibrils were subsequently dissolved in SDS-PAGE sample buffer and loaded on 15% SDS-PAGE. The phosphorylated form of myosin regulatory light chain (RLC) was detected with phosphospecific RLC antibodies (produced earlier [23]), which recognize the phosphorylated form of the RLC followed by a secondary goat anti-rabbit antibody conjugated with the fluorescent dye, IR red 800. The total RLC protein was detected with polyclonal RLC CT-1 antibodies produced in this laboratory [19] (raised against 15 residues from the C-terminus of human cardiac RLC) and served as a loading control. Mouse cardiac myofibrils from LV and RV were also used to determine sarcomeric protein phosphorylation by ProQ/Coomassie [17]. After separation of the samples on 15% SDS-PAGE ProQ Diamond phosphoprotein gel stain reagent (Invitrogen) was used (as described in the manufacturer's manual) to assess phosphorylation of troponin (TnT, TnI) and myosin RLC. The total protein was further detected in the same gel using the Coomassie brilliant blue staining. Myofilament protein phosphorylation ratio (ProQ) was calculated relative to the corresponding Coomassie brilliant blue staining (ProQ/Coomassie) using Image J software.

### 2.7. Statistical Analysis

All values are shown as means ± SEM (standard error of the mean). Statistically significant differences between two groups were determined using an unpaired Student's *t*-test, with significance defined as *P* < 0.05. Comparisons between multiple groups were performed using one-way ANOVA (Sigma Plot 11; Systat Software, San Jose, CA). Passive tension measurements were analyzed using one-way repeated measures ANOVA (IBM SPSS statistics version 21).

## 3. Results

### 3.1. Histology

**Figure 1:**
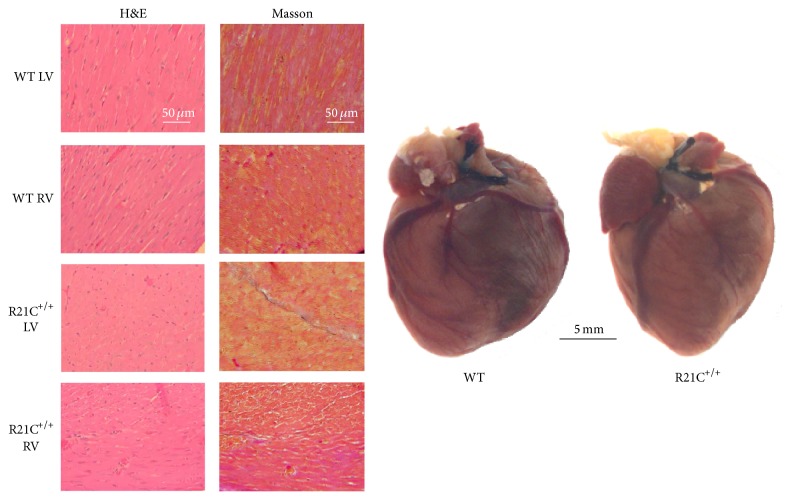
Representative hearts and H&E and Masson's trichrome-stained LV and RV sections from R21C^+/+^ and WT mice. LV and RV sections from the hearts of ~7-month-old male R21C^+/+^ and WT mice were imaged. Note: no mutation-induced abnormalities in H&E stained sections from the hearts of R21C^+/+^ compared with WT mice. Very mild histopathological changes could infrequently be observed in Masson's trichrome-stained LV sections from R21C^+/+^ mice.

Representative H&E and Masson's trichrome-stained LV and RV sections from the hearts of ~7-month-old male R21C^+/+^ and WT mice and images of the whole hearts are presented in Figure 1. The heart tissue morphology pictured in H&E stained slides showed no mutation-induced abnormalities in LV or RV of R21C^+/+^ mice. Very mild histopathological changes could occasionally be seen in Masson's trichrome-stained LV sections from R21C^+/+^ mice compared to WT controls, but no obvious signs of fibrosis or myofilament disarray were observed. These results are in accord with previous findings on R21C^+/+^ mice showing no abnormalities in the hearts of 3–6-month-old mutant versus WT mice [14]. Substantial morphological changes with severe fibrotic lesions were observed in animals as old as 18 months [14].

### 3.2. The R21C Mutation in cTnI Imposes Differences in Maximal Steady-State Force in the LV but Not in RV

**Figure 2:**
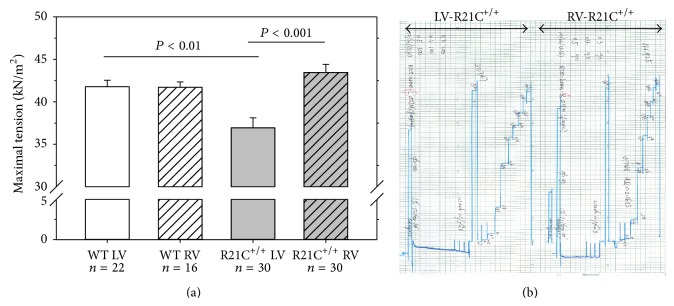
The effect of the HCM-R21C mutation in cTnI on steady-state force development in LV versus RV papillary muscle strips in genetically altered mice. (a) Maximal tension per cross-section of muscle strip. Note that the R21C mutation in cTnI imposes differences in maximal steady-state force in the LV but not in RV. Sixteen to thirty independent measurements on skinned cardiac muscle strips from the left and right ventricles of three WT hearts and four homozygous R21C^+/+^ knock-in hearts have been performed. ~8-month-old mice were used yielding, respectively, *n* = 22 and *n* = 16 muscle strips from the LV and RV of WT mice and *n* = 30 muscle strips from LV and RV of R21C^+/+^ mice. Error bars are SEM. (b) Representative force traces in the LV and RV papillary muscle fibers of R21C^+/+^ mice.

Measurements of steady-state force were performed in skinned papillary muscle strips from the LV and RV of R21C^+/+^ KI homozygous mice and the results were compared to WT mice (Figures 2(a) and 2(b)). Three to four hearts per group were used with each heart yielding 5–8 muscle strips from LV and RV that were used in mechanical experiments (Table 1). A significantly lower maximal isometric force was observed in LV of R21C^+/+^ mice (36.9 ± 1.2 kN/m^2^, *n* = 30 strips) compared to LV-WT (41.8 ± 0.8 kN/m^2^, *n* = 22) strips (Figure 2 and Table 1). The differences in the level of force in LV- R21C^+/+^ versus all other muscles including RV- R21C^+/+^ (43.5 ± 1.0 kN/m^2^, *n* = 30) were also statistically significant (Figure 2, *P* < 0.001). There were no differences between WT-LV and WT-RV (41.7 ± 0.6 kN/m^2^, *n* = 16) or RV- R21C^+/+^ (Figure 2(a) and Table 1). Therefore, the mutation exerted its effect only on the LV and not RV while there were no differences between ventricles of WT mice.

### 3.3. Force-pCa Relationship Is Rightward Shifted in the LV but Leftward Shifted in RV in R21C^+/+^ Compared to WT Mice

**Figure 3:**
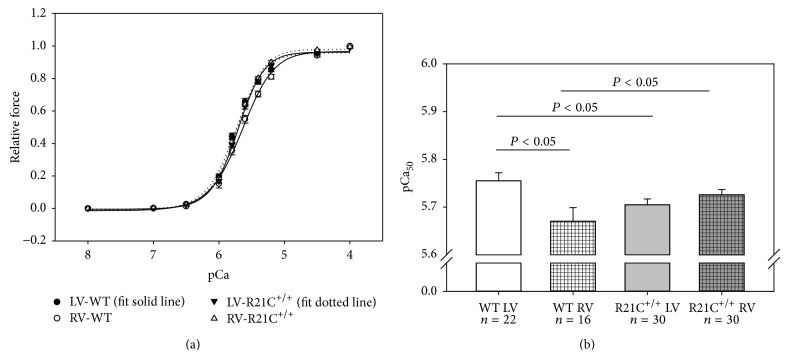
Force-pCa dependence (a) and Ca^2+^-sensitivity (b) in LV and RV of R21C^+/+^ mice compared with LV and RV of WT mice. Note that the Ca^2+^ sensitivity of force was lower in RV-WT compared to LV-WT (*P* < 0.05). The Ca^2+^ sensitivity of force was also lower in the LV of R21C^+/+^ mice versus LV-WT. The pCa_50_ was larger in the RV-R21C^+/+^ compared to RV of WT mice (*P* < 0.05). No differences in the force-pCa dependence were observed between the LV versus RV of R21C^+/+^ mice. Number of mice and muscle strips are as in Figure 2. Error bars are SEM.

As shown in Figures 3(a) and 3(b), there was a significant difference in the Ca^2+^ sensitivity of force between LV-WT (pCa_50_ = 5.76 ± 0.02, *n* = 22) and RV-WT (5.67 ± 0.03, *n* = 16) muscles and the Ca^2+^ sensitivity of force was rightward shifted in the RV of WT mice compared to LV-WT (Figure 3(b), Table 1). The Hill coefficient (parameter of cooperativity) was also slightly lower in RV-WT (*n*_H_ = 1.90 ± 0.10) compared to LV-WT (*n*_H_ = 2.16 ± 0.14), but the difference was not statistically significant (Table 1). Considering the HCM R21C^+/+^ heart, the mutation exerted no statistically significant changes in the Ca^2+^-sensitivity of contraction between the LV and RV (Figure 3). The HCM phenotype of the R21C mutation was manifested by a small but significant change in the Ca^2+^ sensitivity in the LV-R21C^+/+^ (5.71 ± 0.01, *n*_H_ = 2.19 ± 0.10, *n* = 30) compared to the LV-WT. However, as observed for other HCM mutations, the force-pCa relation was leftward shifted in the RV of R21C^+/+^ mice (5.73 ± 0.01, *n*_H_ = 2.20 ± 0.09, *n* = 30) compared to RV-WT (Figure 3, *P* < 0.05).

### 3.4. The R21C Mutation in cTnI Imposes Differences in Passive Force in the LV but Not in RV

**Figure 4:**
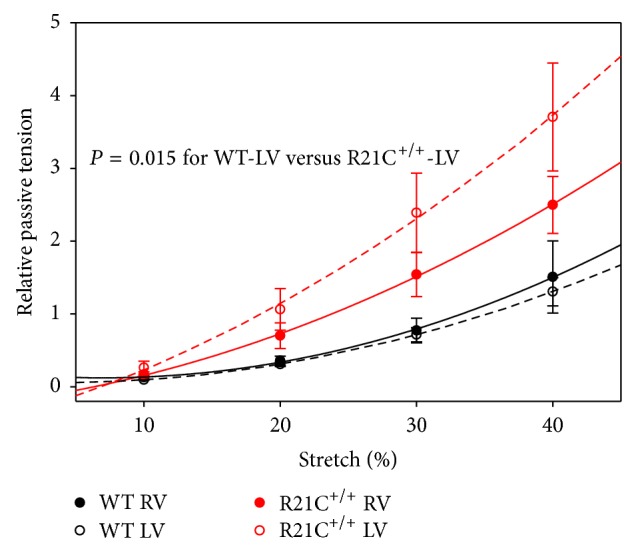
Passive tension per cross-section of muscle strip in LV and RV of R21C^+/+^ mice compared with LV and RV of WT mice. After skinning the strips were washed in the relaxing solution and their length was adjusted to remove the slack to sarcomere length (SL) = ~2.1 *µ*m. This point was set as zero for both the passive force and starting length of the muscle strip. Then, the strips were stretched by 10% of its length ×4 consecutive times, and the passive force per cross-section of muscle (in kN/m^2^) was determined. Note that the R21C mutation exerted its effect on passive tension in the LV compared to LV-WT (*P* = 0.015 as established by repeated measures ANOVA). The values of passive tension (in kN/m^2^) for 10%, 20%, 30%, and 40% stretch were 2.2 ± 0.4; 7.9 ± 0.7; 19.6 ± 1.8; and 38.7 ± 3.5 for LV-WT and 6.4 ± 2.1; 26.6 ± 7.5; 59.7 ± 15.5; and 92.1 ± 21.7 for LV-R21C^+/+^. No statistical differences in the resistance to stretch were observed between the LV versus RV of WT mice. Interestingly, the passive tension for 10% stretch (in kN/m^2^) was 2.2 ± 0.4 (LV-WT); 2.9 ± 0.5 (RV-WT); 7.1 ± 2.2 (LV-R21C^+/+^); and 4.4 ± 1.0 (RV-R21C^+/+^). The difference between LV-mutant and LV-WT was statistically significant (*P* = 0.027) indicating mutation-induced partial loss of Ca^2+^ regulation at low Ca^2+^. Data are average of *n* = 9-10 experiments performed on skinned muscle strips from 9-month-old male mice. Error bars are SEM.

The measurement of passive tension in response to muscle stretch was performed in pCa 8 relaxing solution as described in [16]. The data are expressed as fold change over the active force measured in kN/m^2^ in pCa 4 solution before the first 10% stretch of the fiber length (Figure 4). Data are average of 9-10 experiments performed on skinned papillary muscle strips from flash frozen hearts of 9-month-old male mice. The effect of mutation on passive tension followed the trend observed in active tension development presented in Figure 2. Passive tension was significantly higher in LV-R21C^+/+^ compared to LV-WT for all points of stretch (Figure 4, *P* = 0.015). No significant changes in the resistance to stretch were noted for other groups of strips. The level of passive tension for 10% stretch (in kN/m^2^) was 2.2 ± 0.4 (LV-WT); 2.9 ± 0.5 (RV-WT); 7.1 ± 2.2 (LV-R21C^+/+^); and 4.4 ± 1.0 (RV-R21C^+/+^). Interestingly, the difference between LV-R21C^+/+^and LV-WT was statistically significant (*P* = 0.027) indicating partial loss of Ca^2+^ regulation at low [Ca^2+^] in the mutant LV. Therefore, once again, it is important to note that the effect of a disease causing mutation was manifested in the papillary muscle strips from the LV (and not RV), the ventricle which is predominantly affected by HCM disease.

### 3.5. The R21C Mutation Prevents cTnI Phosphorylation in Both Ventricles but Imposes Differences in RLC Phosphorylation between the LV and RV

**Figure 5:**
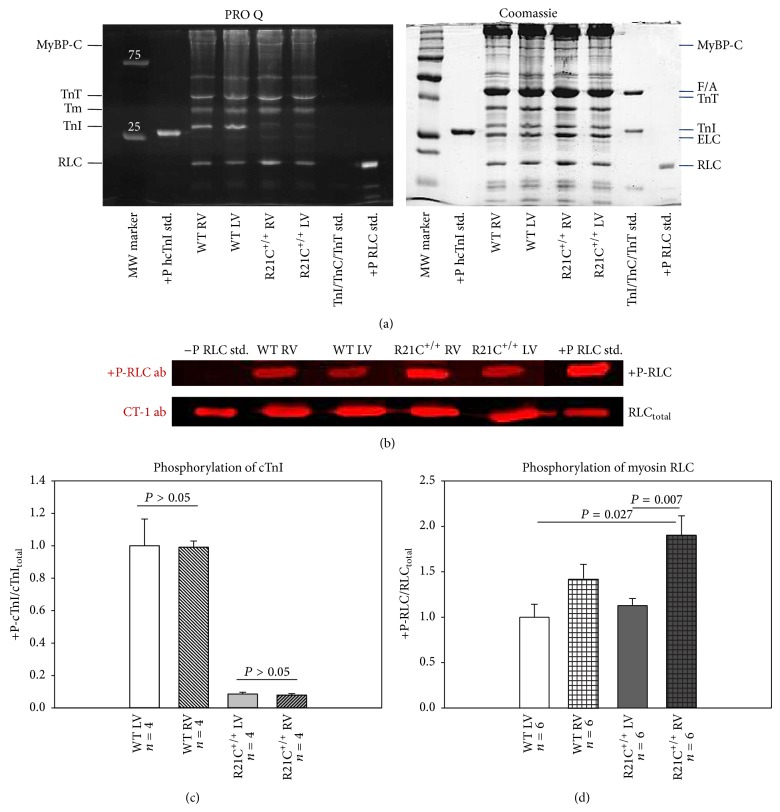
Assessment of protein phosphorylation in LV and RV of R21C^+/+^ mice compared with LV and RV of WT mice. (a) Representative ProQ/Coomassie gels of myofibrillar preparations from LV and RV of R21C^+/+^ mice compared with LV and RV of WT mice. MyBP-C, myosin binding protein C; F/A, F-actin; TnT, troponin T; Tm, tropomyosin; TnI, troponin I, ELC, myosin essential light chain; RLC, myosin regulatory light chain; +P hcTnI std., phosphorylated human cardiac TnI standard; and +P RLC std., phosphorylated human cardiac RLC standard. (b) Representative Western blot of myofibrillar preparations from LV and RV of R21C^+/+^ mice compared with LV and RV of WT mice. The level of RLC phosphorylation was determined with phosphospecific RLC antibodies (+P-RLC ab) and compared to the total RLC content assessed with a rabbit polyclonal RLC antibody (CT-1 ab) recognizing total RLC protein. −P RLC std., nonphosphorylated RLC and +P RLC std., phosphorylated RLC standard proteins. (c) Quantification of phosphorylated cTnI was assessed by *n* = 4 independent SDS-PAGE (ProQ/Coomassie gels) analyses of two to three preparations from LV and RV ventricles per group. (d) Quantification of RLC phosphorylation was assessed by *n* = 6 SDS-PAGE analyses (Western blots and ProQ/Coomassie gels) of two to three LV and RV preparations from WT and R21C^+/+^ mice. Note no cTnI phosphorylation in R21C^+/+^-KI mouse model. There were no differences in cTnI phosphorylation between the LV and RV of WT mice. Note significantly enhanced phosphorylation in the RV-R21C^+/+^ compared to LV-R21C^+/+^ (*P* = 0.007) and to LV-WT (*P* = 0.027). No statistically significant differences in myosin RLC phosphorylation were observed between the LV and RV of WT mice. Errors bars are SEM.

As shown in Figures 5(a) and 5(c), there were no differences in cTnI phosphorylation between the LV and RV of WT mice. Consistent to what was observed earlier in this R21C^+/+^-KI mouse model of HCM [14, 15], the mutation prevented *β*-adrenergic-activated protein kinase A- (PKA-) mediated phosphorylation of cTnI at Ser 23 and 24. Data were derived from four independent ProQ/Coomassie gels assessed in 2-3 preparations from LV and RV of R21C^+/+^ and WT mice. No changes in phosphorylation of troponin T (TnT) or myosin binding protein C (MyBP-C) were observed between the groups of fibers (Figure 5(a)). However, the mutation imposed a significant alteration in the myosin RLC phosphorylation between the LV and RV of R21C^+/+^ mice (Figures 5(b) and 5(d)). Data for RLC phosphorylation were derived from six independent SDS-PAGE (Western blots and ProQ/Coomassie gels) analyses of two to three LV and RV preparations per group. Interestingly, phosphorylation of myosin RLC was significantly higher in the RV versus LV of R21C^+/+^ mice (Figures 5(b) and 5(d), *P* = 0.007) and also significantly higher than in LV-WT (Figures 5(b) and 5(d), *P* = 0.027). Elevated RLC phosphorylation in the RV of R21C^+/+^ mice may play rescuing role in preventing the right ventricle from abnormalities in force development that are observed in the LV of R21C^+/+^ mice. No statistically significant differences in myosin RLC phosphorylation were observed between the LV and RV of WT mice (Figure 5(d), *P* > 0.05).

## 4. Discussion

In this study, we aimed to examine the effect of the HCM-linked R21C in cTnI on the function and protein phosphorylation and pinpoint potential differences between the LV and RV using papillary muscle fibers from the KI homozygous mice compared to WT. The R21C mutation was identified in a cardiomyopathy patient, who presented with atrial fibrillation shortly after the sudden death of her child at the age of 18 years [13]. Three surviving mutation carriers from the family had asymmetrical septal hypertrophy, left atrial enlargement, and normal cardiac dimensions. The mutation was also identified in another HCM family with 4 mutation carriers having subaortic asymmetrical hypertrophy and one mutation carrier with normal cardiac dimensions, who was resuscitated from sudden death [13]. The animal model of R21C-HCM used in this study was produced and characterized previously [14, 15]. In agreement with those reports, we show that the mutation renders no obvious histopathological changes in ~7-month-old mice compared to age and gender matched WT (Figure 1). Likewise, as shown earlier, we observed no cTnI phosphorylation in R21C^+/+^ mice (Figure 5). However, we show here that the R21C mutation in cTnI imposes significant functional differences in contractile force generation in genetically altered KI-R21C^+/+^ compared to WT mice. In contrast to Wang et al., 2012 [14], a significantly lower maximal pCa 4 force was observed in R21C^+/+^ compared with WT mice (Figure 2). Importantly, this difference in maximal tension per cross-section of muscle occurred only in the LV and not in the RV of R21C^+/+^ mice (Figure 2). This result highlights the importance of testing cardiac muscle preparations from both ventricles when studying the effect of HCM causing mutations in mice. Interestingly, passive tension in R21C^+/+^ mice followed the active force measurements and the only difference between R21C^+/+^ and WT fibers was observed in the LV. A significantly larger value for passive tension was observed in LV-R21C^+/+^ compared to LV-WT (Figure 4, *P* = 0.015). No statistically significant changes in the resistance to stretch were noted for other groups of muscle strips. In addition, the value of passive tension for 10% stretch was 3.2-fold larger in LV-R21C^+/+^ than in LV-WT (*P* = 0.027). Elevated passive tension measured in the LV of R21C^+/+^ versus WT mice indicated partial loss of Ca^2+^ regulation at low [Ca^2+^]. Therefore, once again, it is important to note that the effect of a disease causing mutation was manifested in the papillary muscle strips from the LV (and not RV), the ventricle which is predominantly affected due to HCM disease.

There are various functional differences between the LV and RV of WT mice that could arise from differences in external loads of both ventricles, differently occurring posttranslational modifications, and so forth, and be manifested by changes in the myofilament Ca^2+^ sensitivity between LV and RV. In agreement with the study by Perreault et al. [24] and Belin et al. [11], we observed a rightward shift in the force-pCa curve in the RV versus LV of WT mice (Figure 3). There were no differences in the Ca^2+^ sensitivity between the RV and LV of R21C^+/+^-KI mice. In R21C^+/+^ mice, the R21C mutation disrupts the PKA consensus sequence preventing PKA-dependent phosphorylation of Ser 23/24 of TnI that occurs in response to *β*-adrenergic stimulation in WT mice resulting in faster relaxation and desensitization of myofilaments to calcium [25–28]. As seen for the majority of HCM causing mutations, especially for the regulatory troponin proteins [29, 30], the R21C mutation left shifted the Ca^2+^ sensitivity of force but only in the RV compared to RV-WT. The HCM phenotype in the LV of R21C^+/+^-KI mice was manifested by a rightward shift in the Ca^2+^ sensitivity of force compared to LV of WT mice. A mutation-elicited response in the LV of R21C^+/+^ mice was similar to that observed on *β*-adrenergic stimulation causing desensitization of myofilaments to calcium. However, the latter did not occur due to the PKA-dependent phosphorylation of TnI, which was absent in R21C^+/+^ mice. Regarding the phenotypes between the LV and RV, the mutant showed a significantly lower Ca^2+^ sensitivity (pCa_50_) than WT in the LV, while it resulted in larger pCa_50_ than WT in the RV (Figure 3(b)). In conclusion, the HCM phenotype in R21C^+/+^ mice was manifested by the lack of differences in the Ca^2+^ sensitivity of force between the LV and RV that were clearly observed in the ventricles of WT mice.

To elucidate the reason for contractile differences (in force and calcium sensitivity) observed in R21C^+/+^ versus WT mice, we have examined sarcomeric protein phosphorylation in the LV and RV of mice (Figure 5). The phosphorylation level of cTnI was not different between the LV and RV of WT mice, while no phosphorylation of cTnI was seen in LV-R21C^+/+^ and RV-R21C^+/+^ (Figures 5(a) and 5(c)). As observed in this study, the R21C mutation was shown before to prevent the PKA-dependent phosphorylation of Ser 23 and Ser 24 of cTnI [14, 29]. In addition, no mutation exerted changes were observed in phosphorylation of TnT or MyBP-C (Figure 5(a)). However, a significant R21C-mediated enhancement of myosin RLC phosphorylation was observed in the RV compared with LV of R21C^+/+^-KI mice (Figures 5(a), 5(b), and 5(d)). Cardiac myosin RLC is a major regulatory subunit of muscle myosin and a modulator of the Ca^2+^-controlled regulation of muscle contraction [31]. It is localized at the head-rod junction of the myosin heavy chain and it contains the myosin light chain kinase- (MLCK-) specific phosphorylatable Ser15. The level of RLC phosphorylation has been shown by us and others to play a critical role in cardiac muscle contraction and in the Ca^2+^-sensitive interaction of myosin cross-bridges with the actin-containing thin filaments [32–35]. Largely reduced RLC phosphorylation was reported in patients with heart failure [36, 37] and observed in animal models of cardiac disease [38–41]. Increased myosin RLC phosphorylation was also observed as a preventive measure of cardiac hypertrophy in mice [42]. Since no significant differences in RLC phosphorylation were noted between the LV and RV of WT mice and LV of R21C^+/+^ mice (Figure 5(d)), one can speculate that elevated RLC phosphorylation in the RV of R21C^+/+^ mice may play a rescue role alleviating or preventing mutation-induced contractile abnormalities and maintaining the RV tension at the level of WT. Our results indicate that the lower level of RLC phosphorylation in the LV versus RV of R21C^+/+^-KI mice may contribute to the HCM phenotype that can be clearly observed in the LV and not in the RV of R21C^+/+^ mice.

## Figures and Tables

**Table 1:** The effect of R21C mutation in TnI on steady-state force measurements in KI R21C^+/+^ mice.

Parameter	LV-WT mice	RV-WT mice	LV-R21C^+/+^ mice	RV-R21C^+/+^ mice
	Three mice; 22 fibers	Three mice; 16 fibers	Four mice; 30 fibers	Four mice; 30 fibers
Maximal tension/cross-section (kN/m^2^)	41.8 ± 0.8	41.7 ± 0.6	36.9 ± 1.2	43.5 ± 1.0
pCa_50_ (calcium sensitivity)	5.76 ± 0.02	5.67 ± 0.03	5.71 ± 0.01	5.73 ± 0.01
*n*_H_ (Hill coefficient)	2.16 ± 0.14	1.90 ± 0.10	2.19 ± 0.10	2.20 ± 0.09
